# A Randomized Study Comparing Skin Staples with Subcuticular Sutures for Wound Closure at Caesarean Section in Black-Skinned Women

**DOI:** 10.1155/2014/807937

**Published:** 2014-10-28

**Authors:** Rukiyat Adeola Abdus-Salam, Folasade Adenike Bello, Oladapo Olayemi

**Affiliations:** ^1^Department of Obstetrics and Gynaecology, Adeoyo Maternity Teaching Hospital, Yemetu, Ibadan 200211, Nigeria; ^2^Department of Obstetrics and Gynaecology, College of Medicine, University of Ibadan, Ibadan 200212, Nigeria; ^3^Department of Obstetrics and Gynaecology, University College Hospital, PMB 5116, Ibadan 200212, Nigeria

## Abstract

This study aimed to compare patients' satisfaction and outcome of caesarean section wound closure by skin staples and subcuticular suture at discharge and 6 weeks of postoperation. It was a randomized controlled trial of pregnant women scheduled for caesarean section at the University College Hospital, Ibadan, Nigeria, allocating them to wound closure by skin staples or subcuticular suture. Pain was assessed using the box numeric pain scale. Scar assessments were by patient, research nurse, and independent observers using the visual analogue scale, modified patient observer scar assessment scale, and patient satisfaction scale. Operation time (minutes) was significantly shorter in the staple group, 40.26 (±16.53) compared to 47.55 (±14.55) in the suture group (*P* = 0.025). Skin closure time (seconds) was significantly less in the staple group, 118.62 (±69.68) versus 388.70 (±170.40) in the suture group (*P* ≤ 0.001). There was no difference in pain experienced, wound assessment by the participants, and patients' satisfaction. Participants in the staple group scored higher on both scar assessment scales by the nurse (*P* = 0.044). Cost comparison analysis showed that staple use costs significantly more than suture use (*P* < 0.001). The perceived benefit of subcuticular suture over skin staples was not observed and participants were satisfied with both wound closure techniques.

## 1. Introduction

Caesarean section (CS) is one of the oldest and commonest major surgical procedures, with several million women undergoing caesarean delivery each year [1–4]. The incidence of CS has increased around the world for varying indications. This rise may be due to increased incidence of multiple gestations, decreased attempt at vaginal birth after CS, vaginal breech deliveries, and maternal request [5, 6]. “The role of caesarean section has been transformed in a little more than a century from a procedure of desperation performed only in the rarest and most terrible circumstances to a common place procedure frequently applied especially in affluent societies for what some would regard as trivial indications” [3].

The subsequent apposition of wounds following surgical incision is important to wound healing by primary intention and to reduce postoperative morbidities. The wound closure materials have evolved over the years, varying in caliber, biochemical composition, constituent, knot security, elasticity and absorption, tensile strength, and tissue reactivity [7]. Some of the methods for skin closure at caesarean section include absorbable and nonabsorbable sutures, stainless steel staples (metal staples), absorbable staples, adhesive closure strips, and tissue adhesives [7, 8]. An ideal wound closure device or technique should be easy to use, fast, and painless, provide excellent cosmetic appearance, and be cost-effective [9]; no wound closure device is ideal for all situations and the physician decides which one material best suits the particular closure [7].

The techniques of CS have evolved and have been improved upon over the century; evidence-based refinement has been made to surgical techniques of CS following results from several studies. However, the technique of skin closure with best outcome for CS is poorly studied; and the best method for skin closure at CS is unknown at present [9, 10]. Two Cochrane reviews concluded that there was not enough evidence to state whether any particular technique for closing the abdominal wall at caesarean section is better than others [9, 11]. A study comparing cosmetic outcome of skin closure methods following CS (staples and sutures) found no long term difference in cosmetic outcome between stapled wound and subcuticular suture closed wounds [2], while another study found skin incisions closed with the subcuticular closure to have more cosmetically appealing wounds at postoperative visit compared with skin closed with staples [12, 13]. However, the operating and wound closure time was found to be significantly less with staple skin closure than with subcuticular skin closure [4, 12, 14, 15]. The authors did not find any study comparing outcome of metal staples and sutures in blacks or comparing the cosmetic outcome or scar appearance between black and white skinned women nor on the effect of skin closure technique on wound healing and postoperative pain in black population. Therefore, this study was set to determine the effect of skin closure technique on wound outcome in a black population, comparing skin incision closure with metallic stainless steel staples and subcuticular absorbable suture.

## 2. Methodology

This study was a randomized controlled trial conducted at the University College Hospital, Ibadan, Nigeria, from November 2011 to May 2012. A total of 106 participants were enrolled into the study with fifty-three participants in each study group using a 5% level of significance, power of 80%, and a 10% attrition rate. The subjects included pregnant women who had received antenatal care and delivery at the University College Hospital and had been scheduled for caesarean section for either maternal or foetal indications or both. The eligible participants were counselled and enrolled into the study after an informed consent was obtained. A computer generated table of random numbers was used. The participants were randomized into subcuticular suture or metal skin staple group and the allocations concealed. The patients, surgeons, and the nurse were not blinded to the type of skin closure technique used. It was ensured that the participants or surgeons were not aware of the skin closure material to be used prior to skin closure at surgery. However, the independent observers were blinded to the intervention groups.

The inclusion criteria were caesarean section performed under regional anaesthesia in patients ≥18 years of age and use of Pfannenstiel incision. The exclusion criteria were women with diabetes mellitus, anaemia, HIV infection, skin allergy and other allergies, previous keloid formation or hypertrophic scar, tattoo in skin area of study, chronic steroid use, and maternal risk factor for wound infection—prolonged premature rupture of membranes >24 hours or repeated vaginal examinations (>4).

The patients had routine preoperative preparation and all patients had caesarean section following standard technique of caesarean section after prophylactic perioperative antibiotics. After closure of the rectus sheath, the skin incisions were closed with either metal skin staples or subcuticular absorbable sutures, based on randomization. A trained nurse removed skin staples and assessed the wound at discharge, at removal of staples and at 6 weeks. The B/Braun Manipler AZ-35W (Aesculap, Rubi, Spain) staple device was used. The subcuticular absorbable suture skin closure was done with 2/0 polyglycolic acid suture—Safil (Aesculap, Rubi, Spain). B/Braun (Aesculap, Germany) skin staple remover was used. A visual analogue scale (VAS) [16] was employed for scar assessment. A modified patient and observer scar assessment scale (POSAS) [17], a box numerical pain scale [18], and a patient satisfaction scale were also used. The patient satisfaction scale consisted of a scale of 1–5 ranging between very unsatisfied, unsatisfied indifferent, satisfied, and very satisfied. The duration of skin closure time, subcutaneous fat layer thickness, wound length, and numbers of staple pins used were documented. The pain assessment at stitch site was done on the first, third, and fifth postoperative days, at removal of staples on the tenth day, and at 6 weeks. A VAS was administered to the patients at 5 days, 10 days, and 6 weeks after operation. A scar assessment was also done at 6 weeks using a modified POSAS. The photographs of the wound were taken with a Nikon Coolpix S2550 12.0 megapixel camera at the same camera setting and an approximate distance of 30 cm from the wound for assessment by independent observer. Other parameters included assessment for wound infection, wound separation (partial dehiscence), dehiscence, haematoma, and seroma. The patient's satisfaction was also assessed. The patients were discharged on the fourth or fifth postoperative day according to the unit protocol. The participants with skin wound closure by metal staples returned for staple removal on the tenth postoperative day. The patients' satisfaction was also assessed at six weeks using the patient satisfaction scale.

Cost comparison analysis was done. Caesarean sections are charged at the same amount in the study centre, irrespective of the duration of the procedure, so no differential cost is determined from this. Costing was done by computing the cost of the surgeon's time, the anaesthetist's time, the scrub nurse's time, and cost of closure materials. In addition, for the participants that had staple closure, the cost of their transportation to and from the hospital for staple removal and the nurse's time during removal of staples from the wound were included. No allowance was made for lost income, as they were all on maternity leave at this time.

The cost of a senior registrar's time (divided over regular work hours and on-call hours) was computed as NGN12/minute (USD0.08). This was used as the surgeon's time/minute and anaesthetist's time/minute, respectively (most caesarean sections are done by this level of staff). A similar computation was done for scrub nurse and nurse's time at removal using a rate of NGN10/minute (USD0.06) for a nursing officer's shift-duty (these duties are often done by this level of staff). The cost of wound closure material was NGN500 (USD3.13) for the absorbable suture material and NGN3,000 (USD18.75) for the metal skin disposable stapler and staple remover. The cost of a patient's transportation to return for staple removal was calculated as NGN250 (USD1.56). This was the actual cost of public transportation from a location that was considered to be one of the farthest within the metropolis from the study location. This distance was 11 km, as estimated by Global Positioning System (GPS). All other home distances were assumed to fall within this range. The foreign exchange rate at the time of the study was NGN160 to USD1.

The data were analysed using the Statistical Package for Social Sciences, Chicago II Inc. Version 16. The differences in outcome were compared between the two groups using the chi-square test, Fisher's exact test, and *t*-test. A level of significance of 5% was used for all tests. Ethical approval was obtained from the University of Ibadan/University College Hospital (UI/UCH) Ethics Committee.

## 3. Results

**Figure 1:**
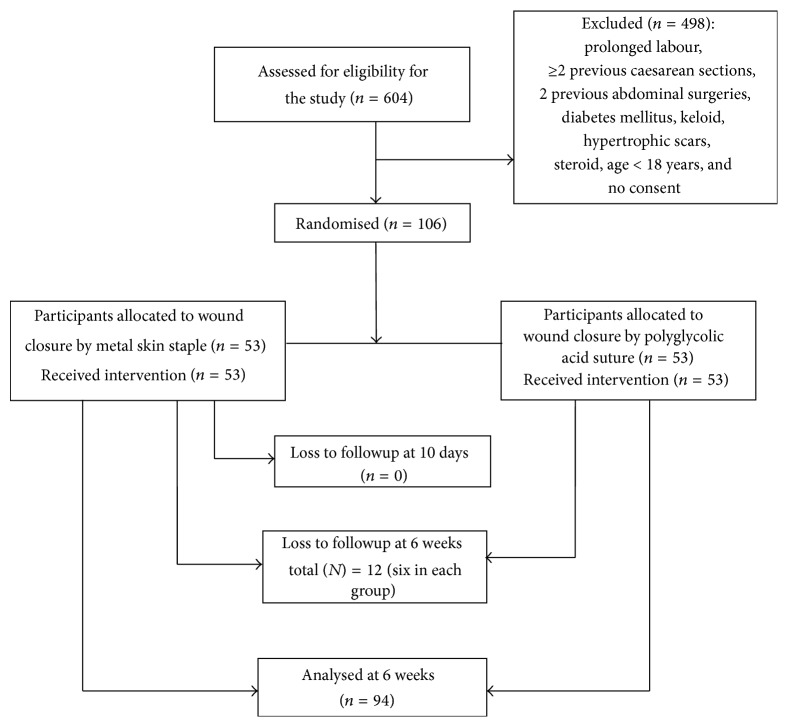
Flow of participants through the study.

The flow of participants through the study is shown in Figure 1. Of the 106 participants recruited, 94 completed the study, 12 were lost to followup and these were excluded from the analysis, forty-seven participants were analysed in each study group. The mean ages of the participants in the skin staple and suture group were 31.6 (±4.50), and 31.1 (±4.27) years, respectively. Tables 1 and 2 show the sociodemographic and obstetric characteristics, respectively, of women in the two groups. There were no differences in the sociodemographic, economic, and obstetric characteristics in the two groups. The difference in the mean weight (Kg) at booking approached significance; this was 69.15 (±13.88) for participants in the staple group and 74.87 ± 14.0 for the suture group (*P* = 0.054). The mean BMI (Kg/m^2^) at booking was 27.04 ± 4.84 for the staple group and 28.25 ± 5.13 for the suture group (*P* = 0.253). Most of the participants were not in labour prior to the caesarean section, forty (85.1%) participants in the staple compared with forty-four (93.6%) participants in the suture group. Seven (14.9%) participants in the staple group compared with three (6.4%) participants in the suture group had uterine contractions at the time of caesarean section and there was no significant difference (Fisher's exact test value = 0.316). These participants had been scheduled for elective caesarean section but started having uterine contractions before the caesarean section and were not in established labour. Twenty (42.6%) of the participants in the staple group and twenty-four (51.1%) of the suture group had one prior caesarean section. Twenty-seven (57.4%) participants in the staple group compared to twenty-three (48.9%) participants in the suture group had a primary caesarean section (*P* = 0.408). The operation time was defined by duration from skin incision to the completion of wound closure. The mean operation time was shorter for participants in the staple group, 40.26 minutes (±16.53) compared to the suture group 47.55 minutes (±14.55) (Table 3) which was statistically significant (*P* = 0.025). The closure time defined by the time from onset to completion of wound closure was also considerably longer for participants in the suture group compared to the staple group (*P* ≤ 0.001). The mean estimated blood loss and wound length for the participants in both staples and suture group were similar.

There was no significant difference in the mean pain score for the participants in either of the study groups (Table 4).  Table 5 shows patients' scar assessment. The mean visual analogue scar assessment score (VAS) of the study participants was similar in the staple group and the suture group on day 5 (*P* = 1.00). The mean score at 6-week assessment was however slightly higher for the participants in the staple group than those in the suture group, though not statistically significant. The assessment of the participants' perception of the wound was done by assessing the scar pain, itchiness, colour, stiffness, thickness, and irregularity. The mean scores and the mean total scores for the participants in the two study groups were similar and none of these were significant. In comparing the mean patient satisfaction scores of the study participants, the mean patient satisfaction scores for the two groups were similar (*P* = 0.452).

Table 6 shows the assessment of the scar by the nurse and the independent observers. The mean VAS score for the participants in the staple group was better than the mean VAS score for the suture group on the fifth postoperative day. This was statistically significant (*P* = 0.023). The difference in the mean VAS score at 6 weeks for the participants in both study groups was not significant (*P* = 0.566). The mean score for the height of the scar at 6 weeks for the participants in the staple group was lower compared to the suture group and this was significant (*P* = 0.041). The lower scores on this scale signify the closeness of the scar to normal skin as possible. The mean total score of the nurse's assessment of the scar of the study participants using the modified observer component of the patient observer scar assessment scale was lower for participants in the staple compared to the suture group (*P* = 0.044). This table also highlights the mean average scores by the independent observers. The mean average visual analogue score and the observer scar assessment score (OSAS) for the participants in both study groups were not significant (*P* = 0.091 and *P* = 0.524), respectively. There was no wound infection or wound dehiscence on day 5. There was no wound complication at 6 weeks.

A cost comparison analysis was done by comparing the mean costs for both wound closure techniques at caesarean section.  Table 7 depicts the cost comparison, for the operation time and the closure time only. Both showed that staple use was significantly more expensive (*P* < 0.001).

## 4. Discussion

The two study groups were comparable for sociodemographic and obstetric characteristics, the body mass index (BMI), and the mean gestational age at booking of the participants. In this study, few participants had previous caesarean section and no stratification was done for primary or repeat caesarean section.

The mean operation time and skin closure time were observed to be shorter in the skin staple group compared to the subcuticular suture group. This is consistent with the findings of other studies by Basha et al. and Rousseau et al. [4, 8].

There is more experience with the use of absorbable subcuticular suture for wound closure at caesarean section compared with skin staples at the study centre. In comparison to the study by Frishman et al. [12] in which the doctors performing the skin closure had more experience with skin staples wound closure than the subcuticular wound closure, the experience with the use of subcuticular suture did not translate to a shorter wound closure time when using absorbable subcuticular sutures. Use of staples will mean a shorter duration of surgery and anaesthesia for the patients and better efficacy of obstetric services in a busy labour ward setting where several patients may require interventions consecutively.

In comparing the mean pain score for the participants in both study groups, there was no difference in the participants' postoperative pain perception and all patients had routine postoperative analgesia. This is contrary to the conclusion of two previous studies; one of the studies showed an increase in pain with the subcuticular suture [4] and the other showed an increase in pain associated with skin staples [12].

There was also no difference in the participants' mean score for assessment of cosmetic wound appearance in both study groups using the visual analogue scale for scar assessment and the patient observer scar assessment scale, similar to other studies [8, 14]. These are subjective pain rating scales and participants' assessment bias could not be excluded. It was also impossible to blind the participants to the wound closure technique used.

Wound assessment by the research nurses however showed that the staple group was significantly more likely to have a higher mean scar assessment score on the fifth day after operation. There was no significant difference in the average VAS score and the modified observer assessment of the patient observer scar assessment scale score by the independent observers for the skin staple and suture groups. The appearance and scar assessment for both groups were similar.

There was also no significant difference in the wound complication at fifth postoperative day and at 6 weeks. However, there were a small proportion of participants with wound seroma in the skin staple group. This compares with findings of other studies with wound complication rates of 1%, 5%, and 7%, respectively [4, 8, 14]. The wound infection rate in previous study on determinants of post-caesarean section wound infection in Ibadan was 16.2% [19] which included elective and emergency caesarean section and patients referred for obstructed labour and complications of labour.

The cost of wound closure technique was significantly higher for the staple group compared to the suture group. This translates to more expense for patients offered metal skin staple relative to patients offered absorbable subcuticular suture which will not require a return clinic visit and removal of the closure material. This suggests that the routine use of staples may not be feasible for low-income-earning patients, especially in settings like the study location where patients pay out of pocket for health care services. For patients that can afford it, however, their satisfaction with the outcome and the shorter operation time may favour this method, despite the cost.

The limitations of the study included the fact that most of the validated scar assessment tools available had components that may be difficult to assess in a black-skinned woman such as vascularity and skin pigmentation; others require instruments for accurate assessment. The tools were therefore appropriately modified. The study was also of short term and did not assess scar maturation over the long term. This may limit the interpretation of its results.

In conclusion, the perceived benefits of the subcuticular suture were not observed in this study. The skin staple was better than the subcuticular suture in terms of scar appearance and there was no difference in pain with either option, making the staple a viable option for use in caesarean section for black women that can afford the extra cost. As patients with comorbidities were not assessed in this study, there may be a need to select such patients carefully until further research is done to assess this.

## Figures and Tables

**Table 1:** Comparison of sociodemographic characteristics of the study participants.

Variable	Staple	Suture		*t* value	*P* value
	Mean (SD)	Mean (SD)			
Age (years)	31.6 (4.5)	31.1 (4.27)		0.517	0.606

Variable	Staples group *N* (%)	Suture group *N* (%)	Total *N* (%)	Chi-square	*P* value

Marital status					1.000^*^
Single	1 (2.1)	1 (2.1)	2 (2.1)		
Married	46 (97.9)	46 (97.9)	92 (97.9)		
Total	**47 (100.0)**	**47 (100.0)**	**94 (100.0)**		
Religion				0.000	1.000
Christianity	36 (76.6)	36 (76.6)	72 (76.6)		
Islam	11 (23.4)	11 (23.4)	22 (23.4)		
Total	**47 (100.0)**	**47 (100.0)**	**94 (100.0)**		
Highest education attained					1.000^*^
Secondary	4 (8.5)	3 (6.4)	7 (7.4)		
Tertiary	43 (91.5)	44 (93.6)	87 (92.6)		
Total	**47 (100.0)**	**47 (100.0)**	**94 (100.0)**		
Monthly income (NGN)^**^				3.641	0.303
<50,000 (<USD312.50)	22 (46.8)	20 (43.5)	42 (45.2)		
≥50,000 (≥USD312.50)	25 (53.2)	26 (56.5)	51 (54.8)		
Total	**47 (100.0)**	**46 (100.0)**	**93 (100.0)**		

^*^Fisher's exact test.

^**^Exchange rate at the time of the study was NGN160 to USD1.

**Table 2:** Comparison of obstetric characteristics of the study participants.

Variables	Stable	Suture		*t* value	*P* value
	Mean (SD)	Mean (SD)			
Gravidity	2.91 (1.49)	3.00 (2.04)		−0.231	0.818
Parity	1.19 (1.23)	1.04 (1.02)		0.640	0.524
GA at booking (weeks)	20.41 (8.59)	20.88 (8.65)		−0.248	0.805
GA at delivery (weeks)	38.39 (2.04)	38.33 (2.17)		0.142	0.887
Weight at booking (kg)	69.15 (13.88)	74.87 (14.00)		−1.956	0.054
BMI at booking (kg/m^2^)	27.04 (4.84)	28.25 (5.13)		−1.151	0.253

Variables	Staples group *N*-47 (%)	Suture group *N*-47 (%)	Total *N*-94 (%)	Chi-square	*P* value

Obstetric risk factor				0.720	0.396
No	20 (42.6)	16 (34.0)	36 (38.3)		
Yes	27 (57.4)	31 (66.0)	58 (61.7)		
Labour before C/S					0.316^*^
No	40 (85.1)	44 (93.6)	84 (89.4)		
Yes	7 (14.9)	3 (6.4)	10 (10.6)		
One previous C/S				0.684	0.408
No	27 (57.4)	23 (48.9)	50 (53.2)		
Yes	20 (42.6)	24 (51.1)	44 (46.8)		

^*^Fisher's exact test value; C/S: caesarean section; GA: gestational age; BMI: body mass index; SD: standard deviation.

**Table 3:** Mean differences between operative variables of the participants.

Variable	Staples group Mean (SD)	Suture group Mean (SD)	*t* value	*P* value
Operation time (minutes)	40.26 (16.53)	47.55 (14.55)	−2.272	0.025
Skin closure time (seconds)	118.62 (69.68)	388.70 (170.40)	−10.058	<0.001
Estimated blood loss (mL)	471.33 (196.86)	484.33 (268.71)	−0.262	0.794
Wound length (cm)	16.68 (2.67)	16.93 (3.63)	−0.388	0.699

**Table 4:** Differences between the mean box numeric pain scores (BNS) of the study participants.

Variable	Staple Mean (SD)	Suture Mean (SD)	*t* value	*P* value
Operation day	5.13 (3.24)	5.62 (3.43)	−0.711	0.479
1st day after operation	4.30 (2.60)	4.00 (2.68)	0.548	0.585
3rd day after operation	2.83 (2.27)	2.74 (2.24)	0.183	0.855
5th day after operation	1.89 (2.31)	2.09 (2.06)	−0.423	0.673
6 weeks after operation	0.26 (0.67)	0.28 (0.74)	−0.145	0.885

**Table 5:** Differences between the mean VAS of the study participants and participants' scar assessment scale scores as assessed by the study participants.

Variable	Staple Mean (SD)	Suture Mean (SD)	*t* value	*P* value
VAS score on 5th day after operation	7.36 (1.77)	7.36 (1.74)	0.00	1.000
VAS score at 6 weeks after operation	9.23 (1.63)	9.04 (1.50)	0.592	0.555
Is the scar painful?	1.21 (0.55)	1.19 (0.45)	0.206	0.838
Is the scar itchy?	1.77 (1.27)	1.70 (1.38)	0.233	0.816
Is the scar colour different from normal?	1.57 (1.08)	1.81 (1.65)	−0.814	0.418
Is the stiffness of scar different from normal skin?	2.09 (1.27)	2.70 (1.96)	−1.816	0.073
Is the thickness of scar different from normal skin?	1.81 (1.10)	1.89 (1.34)	−0.337	0.737
Is the scar more irregular than normal skin?	1.66 (1.56)	2.06 (1.61)	−1.236	0.220
Total patient OSAS score	10.10 (4.13)	11.36 (5.39)	−1.266	0.209
Patient satisfaction score at 6 weeks	4.62 (0.71)	4.51 (0.66)	0.756	0.452

POSAS: patient observer scar assessment scale (patient OSAS).

**Table 6:** Differences between the mean scar assessment scores of the two groups as assessed by the nurse and independent observers.

Variable	Staple Mean (SD)	Suture Mean (SD)	*t* value	*P* value
Nurse VAS score on day 5	7.57 (1.30)	7.00 (1.10)	2.31	0.023
Nurse VAS score at 6 weeks	8.71 (1.45)	8.55 (1.23)	0.58	0.566
Nurse observer scar width (OSAS) score	1.94 (0.67)	2.26 (1.01)	−1.80	0.075
Nurse observer scar height (OSAS) score	1.74 (0.74)	2.09 (0.86)	−2.07	0.041
Nurse observer scar pliability (OSAS) score	2.06 (1.13)	2.36 (1.11)	−1.288	0.201
Total nurse OSAS score+	5.74 (1.87)	6.70 (2.62)	−2.039	0.044
Observer 1 VAS score at 6 weeks	8.00 (0.91)	8.32 (1.00)	−1.62	0.109
Observer 2 VAS score at 6 weeks	5.34 (1.81)	5.91 (1.90)	−1.50	0.136
Average VAS score	6.67 (1.19)	7.11 (1.33)	−1.71	0.091
Observer 1 OSAS width	2.49 (0.72)	2.32 (0.78)	1.10	0.275
Observer 2 OSAS width	4.55 (1.73)	4.26 (1.71)	0.84	0.404
Observer 1 OSAS height	2.26 (0.94)	2.19 (1.10)	0.30	0.763
Observer 1 OSAS height	3.51 (1.49)	3.51 (1.53)	−0.14	0.892
Average OSAS score	6.40 (1.78)	6.15 (1.92)	0.64	0.524

+: modified observer component of the patient observer scar assessment scale (OSAS); VAS: visual analogue scale; modified OSAS: observer scar assessment scale.

**Table 7:** Cost comparison between the staple and suture groups.

Variables	Staples (*N* = 47) Mean (SD)	Suture (*N* = 47) Mean (SD)	*t*-test; *P* value
Cost of operation time, including application +/− removal of closure material (NGN)	4718.68 (561.88)	2082.64 (493.32)	23.716; <0.001
Cost in USD	29.49	13.02	
Cost of wound closure time during surgery and application +/− removal of closure material (NGN)	3417.22 (39.49)	720.26 (96.56)	177.24; <0.001
Cost in USD	21.36	4.50	
